# Detection of Murine Leukemia Virus or Mouse DNA in Commercial RT-PCR Reagents and Human DNAs

**DOI:** 10.1371/journal.pone.0029050

**Published:** 2011-12-20

**Authors:** HaoQiang Zheng, Hongwei Jia, Anupama Shankar, Walid Heneine, William M. Switzer

**Affiliations:** Laboratory Branch, Division of HIV/AIDS Prevention, National Center for HIV/AIDS, Viral Hepatitis, STD, and TB Prevention, Centers for Disease Control and Prevention, Atlanta, Georgia, United States of America; Global Viral Forecasting Initiative, United States of America

## Abstract

The xenotropic murine leukemia virus (MLV)-related viruses (XMRV) have been reported in persons with prostate cancer, chronic fatigue syndrome, and less frequently in blood donors. Polytropic MLVs have also been described in persons with CFS and blood donors. However, many studies have failed to confirm these findings, raising the possibility of contamination as a source of the positive results. One PCR reagent, Platinum Taq polymerase (*pol*) has been reported to contain mouse DNA that produces false-positive MLV PCR results. We report here the finding of a large number of PCR reagents that have low levels of MLV sequences. We found that recombinant reverse-transcriptase (RT) enzymes from six companies derived from either MLV or avian myeloblastosis virus contained MLV *pol* DNA sequences but not *gag* or mouse DNA sequences. Sequence and phylogenetic analysis showed high relatedness to Moloney MLV, suggesting residual contamination with an RT-containing plasmid. In addition, we identified contamination with mouse DNA and a variety of MLV sequences in commercially available human DNAs from leukocytes, brain tissues, and cell lines. These results identify new sources of MLV contamination and highlight the importance of careful pre-screening of commercial specimens and diagnostic reagents to avoid false-positive MLV PCR results.

## Introduction

Xenotropic murine leukemia virus (MLV)-related virus (XMRV) is a new gammaretrovirus reported in persons from the U.S. with prostate cancer or chronic fatigue syndrome (CFS), and in blood donors [1]. MLV-like sequences have also been found in specimens from persons with CFS from the US [2]. However, numerous studies using both PCR and serology, both in the U.S. and abroad, were unable to replicate these findings, stimulating much debate and discussion regarding the origin of the positive results seen in the initial studies [3], [4], [5], [6], [7], [8], [9], [10], [11], [12], [13], [14].

MLVs are endogenous and exogenous retroviruses whose genomes are integrated into mouse chromosomal DNA and can thus be PCR-amplified along with other mouse-specific sequences in specimens contaminated with mouse DNA. Several groups have shown recently that Platinum Taq polymerase from Invitrogen, or reverse transcriptase (RT)-PCR kits containing this enzyme, contain low levels of mouse DNA that produce a positive PCR signal with diagnostic XMRV or MLV primers [1], [4], [15], [16], [17]. The contamination source in Platinum Taq from these studies was linked to carry-over mouse DNA from the mouse monoclonal antibody used to keep Taq inactive during hot-start PCR. XMRV, MLV, and murine sequences have also been found recently in Qiagen nucleic acid extraction columns [18]. High levels of infectious MLV and XMRV have also been found in human cell lines [19], [20], [21]. These results suggest multiple sources of potential contamination of clinical specimens from different cohorts [4], [19], [21], [22], [23], [24].

We report here the identification of widespread MLV contamination of RT enzymes from six manufacturers as well as mouse DNA contamination of commercially available human cell lines and clinical specimens. Our results highlight the importance of careful pre-screening of diagnostic reagents and commercially available specimens to avoid false-positive PCR results during testing of human clinical specimens.

## Results

### Contamination of commercial RT enzymes with MLV plasmid DNA

While investigating the prevalence of XMRV and MLV in persons with CFS and prostate cancer we occasionally detected low levels (<10 copies) MLV and XMRV-like protease (*pro*) sequences in non-template controls (NTC) or negative blood donor plasma by using a quantitative RT-PCR (qRT-PCR) assay that employs the ABI/Ambion AgPath One Step RT-PCR kit (Cat# 4387391, Austin, Tx) [3]. Results for clinical specimens in runs with false-positive negative controls were always considered invalid and were repeated. None of the *pro* sequences detected in the NTC and negative blood donor samples contained the signature sequence present in the positive control template engineered in our laboratory, indicating that the qPCR standard template was not the source of the *pro* signals (data not shown). The recombinant RT enzyme used in the *pro* qRT-PCR testing was included in the ABI/Ambion AgPath One Step RT-PCR kit and according to the manufacturer was derived from an expression plasmid containing the ecotropic Moloney MLV (MoMLV) RT gene. The reagents in this kit are different from those in the Invitrogen One-step RT-PCR kit or Taq enzymes previously found to be contaminated with mouse DNA [1], [4], [15], [17] in that they do not contain mouse monoclonal antibodies to keep the Taq inactive during hot start PCR.

**Figure 1:**
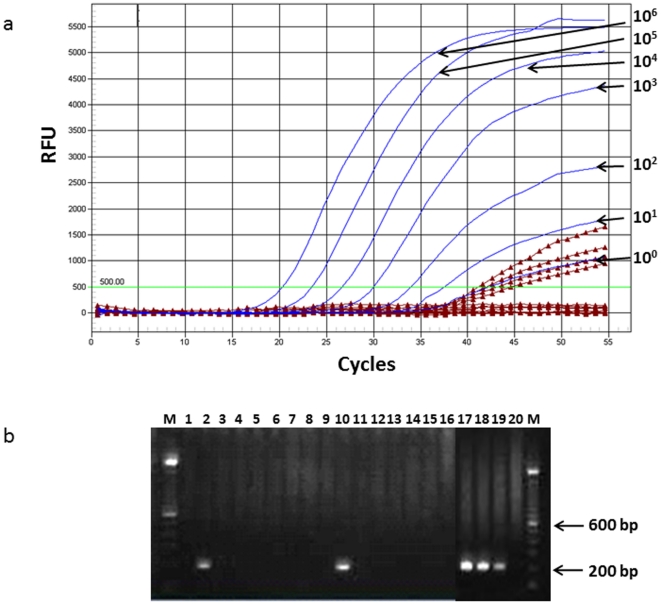
Contamination of commercial AMV reverse transcriptases (RT) with MLV sequences. A. Representative real-time, generic MLV protease (*pro*) amplification plot. Blue lines, XMRV RNA standard extracted from 22Rv1 cell culture supernatants from 10^6^–10^0^ copies per reaction; burgundy lines with triangles, 4/16 (25%) water only controls tested positive for MLV *pro* sequences using the ABI TaqMan Fast 1-step Master Mix; bright green line, RFU, relative fluorescent units. B. Representative gel image showing nested PCR detection of 208-bp MLV polymerase (*pol*) sequences in water only control reactions using Finnzymes RobustI AMV RT. Lanes 1–16, water only controls; lanes 17–20, XMRV RNA extracted from 22Rv1 cell culture supernatants from 10^3^, 10^2^, and 10 copies per reaction, respectively; M. molecular weight marker.

Given the plasmid production history of this enzyme and absence of mouse monoclonal antibodies in the reagents, we suspected that the AgPath RT-PCR kit was contaminated with trace amounts of residual MLV-like plasmid sequences. Furthermore, the enzyme was repeatedly negative for mouse DNA contamination using highly sensitive PCR tests for mitochondrial (mtDNA) and intracisternal A particle (IAP) DNA sequences that can detect attograms of mouse DNA (Table 1) [3]. To evaluate this hypothesis further, we tested multiple replicates of NTC in the *pro* qRT-PCR test. Low levels (Table 1, 1–10 copies/reaction) of MLV/XMRV were found in 7 of 16 replicates in a new, previously un-opened AgPath one step RT-PCR kit. All 16 water-only reactions without the RT enzyme were negative. The proportion of positive NTCs was similar when a larger number of NTC replicates was tested (13/32), but increased significantly when the amount of enzyme was doubled (15/16 positives). Four of five different kit lots tested positive in the *pro* qPCR assay at a frequency ranging from 1/16 to 7/16 replicates when using 1 ul of enzyme per reaction (Table 1). Representative *pro* qPCR results are shown in Fig. 1a. These results document frequent contamination of different lots of this enzyme with low levels of MLV sequences.

**Table 1:** Contamination of commercial RT-PCR reagents with murine leukemia virus (MLV).^1^

*Manufacturer*	*Kit/Reagent*	*RT origin*	*Lot no.*	*pro (%, copies/rxn)*	*gag*	*Nested pol (%)*	*mtDNA* 2 *(copies/rxn)*	*IAP* 2 *(copies/rxn)*
ABI/Ambion	AgPath One Step RT-PCR kit	MoMLV	1009030	7/16 (44, 1–10)	0/16	6/16 (38)	0/16	0/16
	AgPath One Step RT-PCR kit	MoMLV	909020	1/16 (6, 1–10)	ND^3^	ND	ND	ND
	AgPath One Step RT-PCR kit	MoMLV	1009025	2/16 (13, 1–10)	ND	ND	ND	ND
	AgPath One Step RT-PCR kit	MoMLV	1003027	0/16	ND	ND	ND	ND
	AgPath One Step RT-PCR kit	MoMLV	1010031	2/16 (13, 1–10)	ND	ND	ND	ND
ABI	AmpliTaq+AgPath enzyme	MoMLV	1009030	7/16 (44, 1–10)	ND	ND	ND	ND
ABI	TaqMan 1-Step Master mix	MoMLV	1006001	4/16 (25, 1–10)	0/16	12/16 (75)	0/16	0/16
ABI	TaqMan RNA to C_T_ kit	MoMLV	802002	4/16 (25, 1–10)	ND	ND	ND	ND
Agilent	Brilliant II QRT-PCR Master mix	MoMLV	6080963	6/16 (38, 1–10)	0/16	15/16 (94)	0/16	0/16
Promega	Access RT-PCR system	AMV	316238	0/16	ND	ND	ND	ND
	AccessQuick RT-PCR system	AMV	313296	0/16	0/16	0/16	0/16	0/16
	AccessQuick RT-PCR system	AMV	316238	0/16	ND	ND	ND	ND
Roche	Transcriptor 1-step RT-PCR kit	AMV	12275200	8/16 (50, 1–10)	0/16	4/16 (25)	0/16	0/16
	Transcriptor 1-step RT-PCR kit	AMV	12513300	2/16 (13, 1–10)	ND	ND	ND	ND
Finnzymes	RobusT I RT-PCR kit	AMV	127	failed	0/16	2/16 (13)	0/16	0/16

1 The reverse transcriptase (RT) enzymes and kits were tested by quantitative protease (*pro*), polymerase (*pol*), *gag and*/or nested *pol* RT-PCR assays and results shown are number of positive tests out of the total number of replicates. Percentages for replicate testing and copies/reaction (rxn) are in parentheses.

2 Mouse DNA contamination was detected using mitochondrial DNA (mtDNA) and intracisternal A particle (IAP) PCR qPCR assays.

3 ND, not done.

To confirm the contaminant was from a DNA source, we added the AgPath enzyme into a qPCR *pro* DNA PCR test using an AmpliTaq Gold enzyme (Cat # N8080244; ABI, Foster City, CA), previously determined to be free of any MLV/XMRV, and amplified using the same conditions used for the *pro* qRT-PCR test except the first step included a 9 min 95°C step to inactivate the AgPath RT. 7/16 (44%) NTC reactions tested positive (Table 1). The AgPath enzyme was found to be negative for *gag* sequences (0/16), but positive for *pol* sequences (14/16, 88%) using two new qRT-PCR tests to generically detect MLV and XMRV (Table 1).

To investigate the breadth of this contamination in other RT enzymes and kits, we tested both MoMLV- and avian myeloblastosis virus (AMV)-derived recombinant RTs from multiple manufacturers using the *pro* qRT-PCR test or a nested polymerase (*pol*) RT-PCR test that generically detects MLV and XMRV sequences about 216-bp in length using the primers XPOLOF, XPOLOR, XPOLIF, and XPOLOR [5], [9]. AMV and MLV are highly divergent retroviruses in different genera sharing only 28% *pol* nucleotide identity and thus the MLV-based primers and probes should not amplify AMV sequences if present. We found that two MoMLV-based RT kits from ABI (TaqMan 1-step Master Mix and TaqMan RNA to Ct kit; cat #s 43019169 and 432938, respectively, Foster City, CA) and one MoMLV-based kit from Agilent (Brilliant II QRT-PCR Master mix; cat # 600884; Santa Clara, CA) contained low copies of XMRV/MLV sequences in 4/16 (25%)–6/16 (38%) replicates, respectively (Table 1). Likewise, a Roche (Transcriptor 1-step RT-PCR kit; cat # 04655877001; Indianapolis, IN) and a Finnzymes kit (RobusT I RT-PCR kit; cat# F580L; Thermo Fisher Scientific, Lafayette, CO) that both include AMV RT were also positive for XMRV/MLV *pro* (8/16 (50%)) and *pol* sequences (4/16 (25%) and 2/16 (13%), respectively (Table 1). The Finnzymes kit failed to amplify sequences from the standard controls using the *pro* qRT-PCR primers and probes. Thus, the nested *pol* PCR assay was used to test the RT in the Finnzymes kit. Fig. 1b shows the detection of *pol* sequences in 2/16 (12.5%) replicates in the AMV RT from Finnzymes. Increasing amounts of AMV RT from the Finnzymes kit used in the nested *pol* PCR test also yielded increasing quantities of detectable contaminants (5/16, 31%). We also replaced the AMV RT from Finnzymes with an AMV RT from Promega and repeated the testing and found no positive reactions in the water only controls (0/16, data not shown), further supporting the contamination of the Finnzymes RT. In contrast, AMV RTs in two kits from Promega (Access RT-PCR (cat# A1259) and AccessQuick RT-PCR (cat# A1702) systems, Madison, WI) were both negative in all 16 replicates (Table 1). None of the enzymes with detectable MLV sequences were positive for mouse mtDNA or IAP sequences in any of the 16 replicates. These results suggest that the source of the MLV sequences in these enzymes is not mouse DNA.

**Figure 2:**
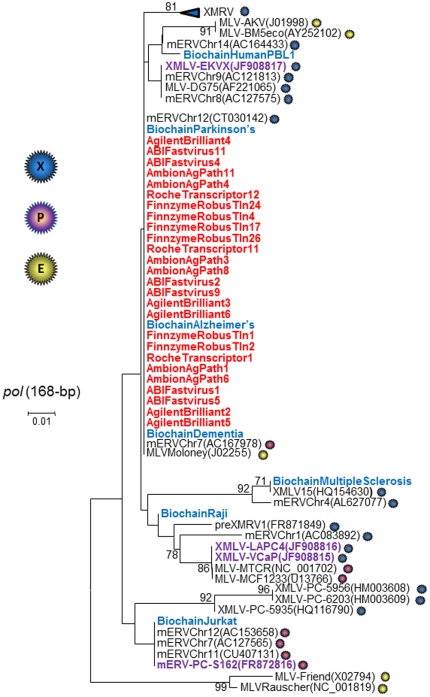
Inference of contamination origin in commercial RT-PCR reagents and human genomic DNAs. Phylogenetic analysis of 168-bp polymerase (*pol*) sequences. Bootstrap values ≥60 are shown. New sequences from the current study are shown in red (RT contaminants) and blue (human genomic DNA contaminants). Sequence names in purple are those reported as contaminants from other human studies. GenBank accession numbers for prototypical murine leukemia virus (MLV) reference sequences are provided in parentheses. XMRV clades are collapsed for presentation only and include identical sequences from CFS-WPI-1106(GQ497344), CFS-WPI-1178(GQ497343), preXMRV2(FR871850), PC-VP35(DQ241301), PC-VP42(DQ241302), PC-VP62(DQ399707), 22Rv1/CWR-R1(FN692043) The 168-bp *pol* sequences are available from the authors upon request. GenBank does not accept sequences less than 200-bp in length. Sequences coded as XMRV VP and WPI are from prostate cancer (PC) and CFS patients, respectively. BD; blood donor sequences. Viral host receptor tropism is indicated by blue (xenotropic), purple (polytropic), and yellow (ecotropic) spheres. PMLV, polytropic MLV; XMLV, xenotropic MLV.

To infer the origin of the contaminants we performed phylogenetic analysis of *pol* sequences from multiple clones from the PCR-positive RT enzymes and kits, multiple prototypic MLV *pol* sequences, and those identified with the highest homology by BLAST analysis using the Neighbor joining distance-based method. The *pol* sequences found in the RT enzymes shared >99% identity with MoMLV (GenBank accession number J02255). Phylogenetic analysis of 168-bp *pol* sequences showed that the RTs and RT kits have MLV-like sequences genetically related to MoMLV that are distinct from XMRV (Fig. 2). The absence of detectable *gag* sequences in the contaminated RT reagents is consistent with a provenance from a plasmid containing only *pol* sequences. As previously reported, the *pol* sequences did not cluster by virus host cell tropism, reflective of a probable history of recombination occurring in this region [3].

### Contamination of commercial human DNAs with mouse DNA

**Figure 3:**
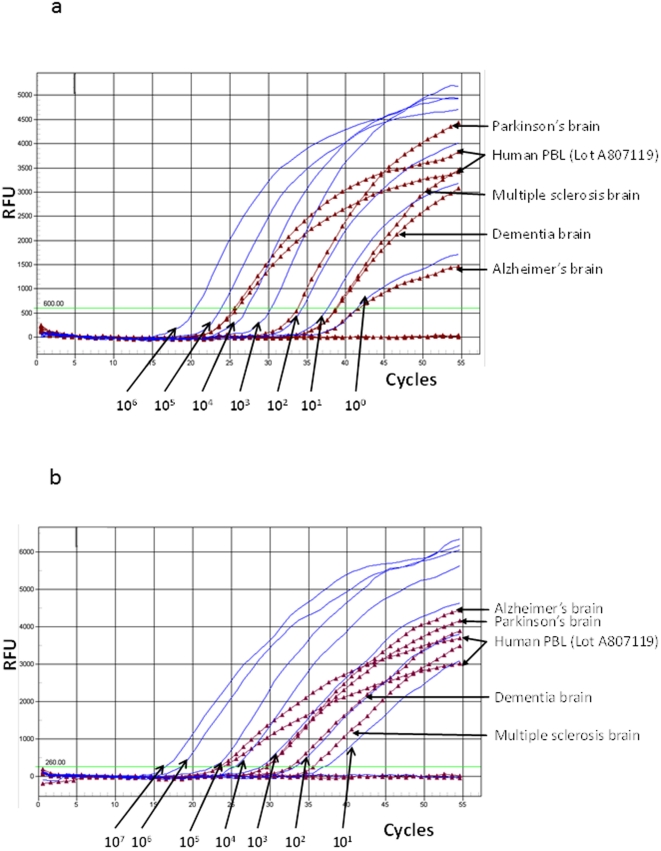
Contamination of commercial human genomic DNA specimens with mouse DNA. A. Representative real-time, generic mouse mitochondrial DNA and B. intracisternal A particle (IAP) polymerase (*pol*) amplification plots. Blue lines, mouse mtDNA or IAP plasmid DNA from 10^6^–10^0^ and 10^7^–10^1^ copies per reaction, respectively; burgundy lines with triangles, human genomic DNA extracted from brain tissue of persons with various inflammatory diseases and two aliquots of the same lot of human peripheral blood lymphocytes (PBL); bright green line, RFU, relative fluorescent units. IAP and mtDNA assay are sensitive to at least 10 copies per reaction.

We also found that commercially available DNAs are contaminated with mouse DNA. Commercially prepared human cell line and tissue DNAs were purchased for use as negative controls in our PCR assays from Sigma-Aldrich (St. Louis, MO) and Biochain (Hayward, CA). All ten Biochain human specimens, including DNA from leukocytes, the B- and T-cell lines Raji and Jurkat, respectively, two tumor cell lines (K562 and MCF-7) and four DNAs from brain tissues of persons with neurologic disorders (dementia, Alzheimer's, Parkinson's, and multiple sclerosis) were positive for XMRV/MLV *pro*, and *pol* sequences, and mouse IAP and mtDNA sequences (Table 2). In addition, all of the Biochain DNAs contained MLV *gag* sequences (Table 2). Although most of the Biochain specimens showed low levels of contamination, the leukocyte DNA had significant amounts of contamination with ∼8,000 copies/µg pro, 39,200 copies/µg IAP, and 232,000 copies/µg mouse mtDNA sequences detected (Table 2, Fig. 3). Although the assays showed the same level of sensitivity using plasmid standards, differences in the amounts of mouse mtDNA versus IAP sequences were detected in some DNA samples and can be explained as pipetting errors or due to increased expression of mtDNA in some cells compared to the stability of integrated IAP sequences. Similar discordant levels of mtDNA and IAP sequences have been reported previously in patient samples contaminated with mouse DNA [22].

**Table 2:** Contamination of commercial human DNAs with mouse DNA.^1^

*Manufacturer*	*DNA Specimen*	*Lot no.*	*pro (copies/rxn)*	*gag*	*pol*	*mtDNA* 2 *(copies/µg)*	*IAP* 2 *(copies/µg)*
Biochain	Human leukocyte DNA	A807119	8,000	Pos	Pos	232,000	39,200
	Human leukocyte DNA^3^	B210149	Neg	Neg	Neg	1–10	1–10
	Dementia DNA	B409046	10	Pos	Pos	46	120
	Parkinson's DNA	B303007	1–10	Pos	Pos	1430	877
	Alzheimer's DNA	B111073	10–20	Pos	Pos	6	1120
	Multiple sclerosis DNA	B409045	1–10	Pos	Pos	52	16
	Raji DNA	NA^4^	Neg	Pos	Pos	58	10–20
	Jurkat DNA	NA	Neg	Pos	Pos	2	10–20
	K562 DNA	NA	Neg	ND	Neg	6	Neg
	MCF-7 DNA	NA	Neg	ND	Neg	Neg	10–20
Sigma-Aldrich	Male placental DNA	88F3847	Neg	Neg	Neg	Neg	Neg
	Female placental DNA	NA	Neg	Neg	Neg	Neg	Neg

1 1 ug of the human DNAs were tested using quantitative (qPCR) *pro* and nested *pol* and *gag* PCR assays. Copies/µg DNA is provided in parentheses for specimens with evidence of contamination.

2 Mouse DNA contamination was detected using mitochondrial DNA (mtDNA) and intracisternal A particle (IAP) PCR qPCR assays.

3 Second lot of human DNA sample received from Biochain after informing them of the contamination results.

4 NA, information not available.

Analysis of the 168-bp *pol* sequences confirmed mouse DNA contamination of the samples with the majority (6/7, 86%) showing 98–100% nucleotide identity to an endogenous retrovirus present on mouse chromosome 12 (GenBank accession # CT030142). The *pol* sequence detected in the MS brain tissue DNA had 99% nucleotide identity to a xenotropic MLV (XMLV15, Genbank accession # HQ154630). In contrast, two human placental DNAs from Sigma-Aldrich both tested negative for XMRV/MLV and mouse IAP and mtDNA sequences (Table 2). Phylogenetic analysis of 168-bp *pol* sequences obtained from the Biochain samples inferred genetic relatedness across the diversity of MLV by clustering with different xenotropic and polytropic MLVs, but which were all distinct from XMRV (Fig. 2).

**Figure 4:**
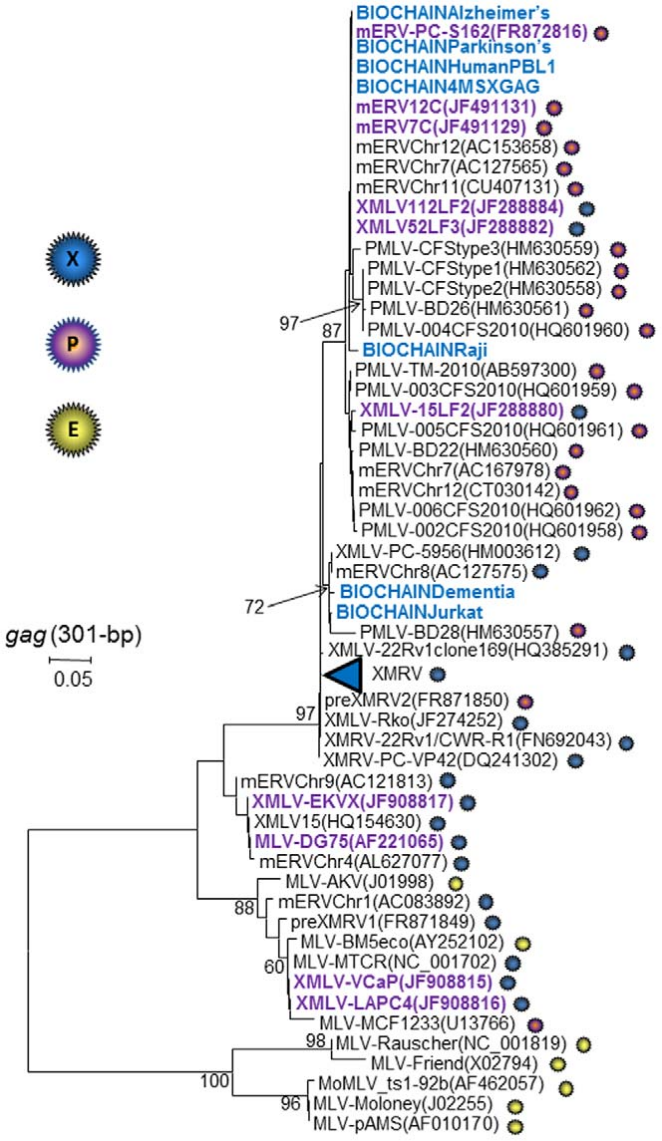
Inference of contamination origin in commercial human genomic DNAs. Phylogenetic analysis of 301-bp *gag* sequences. Bootstrap values ≥60 are shown. New sequences from the current study are shown in blue (human genomic DNA contaminants). Sequence names in purple are those reported as contaminants from other human studies. GenBank accession numbers for prototypical murine leukemia virus (MLV) reference sequences are provided in parentheses. XMRV clades are collapsed for presentation only and include CFS-WPI-1106(GQ497344), CFS-WPI-1178(GQ497343), CFS-WPI-1130(GQ483508), CFS-WPI-1138(GQ483509), CFS-WPI-1169(GQ483510), CFS-WPI-CI-1303(JF907633), CFS-WPI-CI-1313(JF907643), CFS-WPI-CI-1314T(JF907644), CFS-WPI-CI-1307(JF907638), CFS-WPI-CI-1310(JF907641), CFS-WPI-CI-1327(JF907636), preXMRV2(FR871850), PC-VP35(DQ241301),and PC-VP62(DQ399707). Accession numbers for the new *gag* sequences generated in our study are JN629081-JN629087. Sequences coded as XMRV VP and WPI are from prostate cancer (PC) and CFS patients, respectively. BD; blood donor sequences. Viral host receptor tropism is indicated by blue (xenotropic), purple (polytropic), and yellow (ecotropic) spheres. PMLV, polytropic MLV; XMLV, xenotropic MLV.

Likewise, phylogenetic analysis of an alignment of 301-bp *gag* sequences showed that those obtained from the Biochain DNAs were divergent from XMRV and clustered with various xenotropic and polytropic MLVs (Fig. 4). These results are consistent with the genetic diversity of endogenous MLVs in the mouse genome. Interestingly, we also observed the same level of diversity in PMLV sequences identified in CFS patients and blood donors, and in other human specimens and cell lines, congruent with an external mouse cell or DNA contamination source (Fig. 4) [2], [17], [20], [25], [26]. Biochain was notified of these results and independently confirmed the presence of mouse mtDNA sequences in the human leukocyte DNA. After implementing measures to prevent carry-over contamination of human DNAs with mouse material also processed at their facility, a new lot of human leukocyte DNA (B210149) tested negative for mouse mtDNA sequences at Biochain, and MLV sequences at CDC, but was still weakly positive for IAP and mtDNA sequences (1–10 copies/rxn) at CDC (Table 2).

## Discussion

Our results show the frequent contamination of commercial RT enzymes and RT-PCR kits with residual *pol* sequences that are highly related to MoMLV. The absence of detectable MLV *gag* and mouse DNA sequences suggest that the most likely source of the contamination is an expression plasmid vector containing only MoMLV *pro* and *pol* sequences. These results are consistent with patented MoMLV RT-expression vectors that consist of only partial *pro* and 5′ RT sequences. The RNase H and integrase regions of the MoMLV *pol* in this expression vector were selectively removed. The low-level contamination most likely reflects the difficulty in completely removing plasmid DNA with DNase treatment following protein expression. Our data also show that the same MLV sequences are present in AMV RTs, suggesting that the source of contamination of this reagent is not the AMV plasmid but likely carry-over contamination with the MLV RT-containing plasmid. It is possible that such contamination can occur during production of both AMV and MLV RTs in the same laboratories. Although we showed that currently used diagnostic PCR can detect this MLV sequence, the phylogenetic analysis was important to demonstrate that the sequences are highly related to MoMLV, an ecotropic virus that cannot infect human cells, and would thus indicate contamination if found in a human specimen. Our data therefore re-iterate the importance of phylogenetic analysis for all specimens testing PCR-positive [19], [27].

We also demonstrate that commercially prepared human DNAs from a variety of clinical specimens and cell lines can be contaminated with mouse DNA likely originating from mouse tissue processing in the same facility. Unlike the situation observed for the RT kits, we showed that the level of contamination of these specimens can be high and, thus, easy to detect by assays for mouse or MLV DNA. Our findings should serve to remind commercial providers to institute measures to prevent cross-contamination.

### Conclusions

Combined, our results show that MLV and mouse DNA contamination is more widespread in diagnostic reagents and clinical specimens than previously thought. These data reinforce the need for rigorous screening of all diagnostic reagents and specimens by methods capable of detecting trace amounts of MLV and mouse DNA, and to use phylogenetic analysis to assess the biological significance of newly detected sequences. Also, since many of the contaminated RT and PCR reagents are widely used in metagenomics studies, our data call for careful interpretation of any murine retroviral sequences that are detected in these analyses.

## Materials and Methods

### RT-PCR kits and human DNAs

The manufacturer and lot numbers of the nine RT-PCR kits tested in our study are listed in Table 1. Nine human DNAs from either disease tissues or cell lines were purchased from Biochain (Hayward, CA), while the male and female placental DNAs were obtained from Sigma-Aldrich (St. Louis, MO) (Table 2).

### PCR testing for MLVs and mouse sequences

The *pro* qRT-PCR assay was used to screen specimens and reagents for evidence of MLV/XMRV contamination since it generically detects all MLVs and XMRV [3]. This assay uses the Taqman primers Pro-UNV-F1 (5′ CCT GAA CCC AGG ATA ACC CT 3′) and Pro-UNV-R1 (5′ GTG GTC CAG CGA TAC CGC T 3′) and probes Pro-UNV-P1C (FAM5′ AGA TAC TGG GGC CCA ACA CTC CGT GCT GAC 3′BHQ1) and Pro-UNV-PR1 (FAM5′ CCT CCA GTA GCC CCT TGG ACC CAG GC 3′BHQ1) in a one-step RT-PCR reaction mixture at 45°C for 20 min, 95°C for 10 min, followed by 55 cycles of 95°C for 30 sec, 52°C for 30 sec and 62°C for 30 sec. We also use an engineered standard template for quantification of *pro* sequences that contains two unique nine-bp nucleotide insertions that code for the amino acids methionine-leucine-valine (MLV)and tryptophan-methionine-threonine (WMT) separated by a 31-bp hamster gammaretrovirus sequence that are not present in wild-type MLV *pro* sequences [28].

To further evaluate the origin of the MLV sequences detected in the RT reagents and human DNAs we also used three additional PCR tests targeting the *gag* and *pol* regions of the MLV and XMRV genomes. For the *gag* qRT-PCR assay, the primers GAG-UNV-F1, 5′AGGTAGGAACCACCTAGTYC and GAG-UNV-R1, 5′GTCCTCAGGGTCATAAGGAG and probes GAG-UNV-P1C, 5′FAM AGCGGGTCTCCAAAACGCGGGC 3′BHQ1 and GAG-UNV-PR1, 5′FAM CCTTTTACCTTGGCCAAATTGGTGGG 3′BHQ1 were used [5]. For the *pol* qRT-PCR test, the primers POL-UNV-F1 5′CAGAGATGGCTGACTGAGGC3′ and POL-UNV-R1 5′AAACAGAGTCC CCGTTTTGGT3′ and probes POL-UNV-P1C 5′FAM AGGGAGTTCCTAGGGACGGCAGGCTTCT 3′BHQ1 and POL-UNV-PR1C 5′FAM CAAACCCAGGGATCCAGAGGCGACAG 3′BHQ1 were used. Both assays used the same conditions as the *pro* qRT-PCR test and gave sensitivities similar to the *pro* qRT-PCR test detecting 1–10 copies MLV/XMRV per reaction (data not shown). We also used a nested polymerase (*pol*) RT-PCR test that generically detects MLV and XMRV sequences about 216-bp in length using the primers XPOLOF and XPOLOR, and XPOLIF and XPOLOR [5], [9].

For detection of mouse mtDNA, the primers MCOX2F2 (5′ TTC TAC CAG CTG TAA TCC TTA 3′) and MCOX2R1 (5′ GTT TTA GGT CGT TTG TTG GGA T 3′) and probes MCOX2PR1 (5′ FAM-CGT AGC TTC AGT ATC ATT GGT GCC CTA TGG T-BHQ 3′) and MCOX2P1 (5′ FAM-TTG CTC TCC CCT CTC TAC GCA TTC TA-BHQ 3′) were used in a two-step thermocycling of 95°C for 30 sec and 62°C 30 sec for 55 cycles on an iQ5 instrument (Bio-Rad, Hercules, CA). Dilutions of a plasmid containing murine COX2 sequences that was generated by PCR with the MCOX2F1 (5′ ACA TAC AAG CAC AAT AGA TGC 3′) and MCOX2R1 of the murine macrophage cell line RAW264.7 (ATCC, Manassas, VI) were used as the assay standard. For detection of IAP sequences we developed a new qPCR assays that uses the generic primers and probes IAP-MH-POLF2: 5′ GCCTCAYATGTG ATTCAACATTG 3′ and IAP-MH-POLR2: 5′ TTGRGASGTATAWGCTGGT CCATT 3′ and IAP-MH-POL P2: 5′ FAM TTGAGGCMTGGAGTGCTTGGGGRAAACCCAGA 3′BHQ1, respectively. The assay was performed with a hot start at 95°C for 9 min followed by 55 cycles of PCR at 95°C for 30 sec and 62°C for 30 sec. The PCR product amplified from the murine macrophage cell line RAW264.7 DNA with the IAP-MH-POLF2 and IAP-MH-POLR2 primers was cloned and used as the assay standard.

### Preparation of RNA standards used in the RT-PCR testing

XMRV *pol* and *gag* genes were amplified using the primary PCR primers, cloned into pCR2.1 TOPO-vectors (Invitrogen), and transcribed *in vitro* using the Promega RiboMAX large scale RNA production system (T7). Following several DNase treatments to completely remove plasmid DNA, the RNA was quantitated on a Nanodrop spectrophotometer. Copy number was determined using the formula (ng RNA×Avogadro constant)/(RNA length×(1×10^9^)×325) and was verified by comparison of qPCR results using XMRV RNA extracted from 22Rv1 cell culture supernatant quantitated independently [3].

### Sequence analysis

PCR products were purified with QiaQuick PCR or gel purification kits (Qiagen, Valencia, CA) and were directly sequenced on both strands by using ABI Prism BigDye terminator kits and an ABI 3130×l sequencer (Foster City, CA) or following cloning in the TOPO vector (Invitrogen). Initial sequence identity was determined using BLAST analysis at the National Center for Biotechnology Information web server (http://blast.ncbi.nlm.nih.gov/Blast.cgi) using the blastn search option. Sequences were aligned with those retrieved from the BLAST analysis with the highest nucleotide identity, and other MLV prototypes available at GenBank, using MAFFT [29]. Following manual editing and removal of indels, substitution models and phylogenetic relationships were inferred using the neighbor joining (NJ) method implemented in MEGA v5.03 [30]. Support for the branching order was evaluated using 1,000 nonparametric bootstrap replicates. The 168-bp *pol* sequences are available from the authors upon request. GenBank does not accept sequences less than 200-bp in length. Accession numbers for the new *gag* sequences generated in our study are JN629081-JN629087.

## References

[1] Silverman RH, Nguyen C, Weight CJ, Klein EA (2010). The human retrovirus XMRV in prostate cancer and chronic fatigue syndrome.. Nat Rev Urol.

[2] Lo SC, Pripuzova N, Li B, Komaroff AL, Hung GC (2010). Detection of MLV-related virus gene sequences in blood of patients with chronic fatigue syndrome and healthy blood donors.. Proc Natl Acad Sci U S A.

[3] Switzer WM, Jia H, Zheng H, Tang S, Heneine W (2011). No Association of Xenotropic Murine Leukemia Virus-Related Viruses with Prostate Cancer.. PLoS One.

[4] Shin CH, Bateman L, Schlaberg R, Bunker AM, Leonard CJ (2011). Absence of XMRV and other MLV-related viruses in patients with Chronic Fatigue Syndrome.. J Virol.

[5] Satterfield BC, Garcia RA, Jia H, Tang S, Zheng H (2011). Serologic and PCR testing of persons with chronic fatigue syndrome in the United States shows no association with xenotropic or polytropic murine leukemia virus-related viruses.. Retrovirology.

[6] Lintas C, Guidi F, Manzi B, Mancini A, Curatolo P (2011). Lack of Infection with XMRV or Other MLV-Related Viruses in Blood, Post-Mortem Brains and Paternal Gametes of Autistic Individuals.. PLoS One.

[7] Furuta RA, Miyazawa T, Sugiyama T, Kuratsune H, Ikeda Y (2011). No association of Xenotropic Murine Leukemia Virus-related virus with prostate cancer or chronic fatigue syndrome in Japan.. Retrovirology.

[8] Verhaegh GW, de Jong AS, Smit FP, Jannink SA, Melchers WJ (2010). Prevalence of human xenotropic murine leukemia virus-related gammaretrovirus (XMRV) in dutch prostate cancer patients.. Prostate.

[9] Switzer WM, Jia H, Hohn O, Zheng H, Tang S (2010). Absence of evidence of xenotropic murine leukemia virus-related virus infection in persons with chronic fatigue syndrome and healthy controls in the United States.. Retrovirology.

[10] Hong P, Li J, Li Y (2010). Failure to detect Xenotropic murine leukaemia virus-related virus in Chinese patients with chronic fatigue syndrome.. Virol J.

[11] Hohn O, Strohschein K, Brandt AU, Seeher S, Klein S (2010). No Evidence for XMRV in German CFS and MS Patients with Fatigue Despite the Ability of the Virus to Infect Human Blood Cells In Vitro.. PLoS One.

[12] Henrich TX, Li JX, Felsenstein D, Kotton CX, Plenge RX (2010). Xenotropic Murine Leukemia Virus-Related Virus Prevalence in Patients with Chronic Fatigue Syndrome or Chronic Immunomodulatory Conditions.. J Infect Dis.

[13] Cornelissen M, Zorgdrager F, Blom P, Jurriaans S, Repping S (2010). Lack of detection of XMRV in seminal plasma from HIV-1 infected men in The Netherlands.. PLoS One.

[14] Barnes E, Flanagan P, Brown A, Robinson N, Brown H (2010). Failure to Detect Xenotropic Murine Leukemia Virus-Related Virus in Blood of Individuals at High Risk of Blood-Borne Viral Infections.. J Infect Dis.

[15] Sato E, Furuta RA, Miyazawa T (2010). An endogenous murine leukemia viral genome contaminant in a commercial RT-PCR Kit is amplified using standard primers for XMRV.. Retrovirology.

[16] Knox K, Carrigan D, Simmons G, Teque F, Zhou Y (2011). No Evidence of Murine-Like Gammaretroviruses in CFS Patients Previously Identified as XMRV-Infected.. Science.

[17] Tuke PW, Tettmar KI, Tamuri A, Stoye JP, Tedder RS (2011). PCR master mixes harbour murine DNA sequences.. Caveat emptor! PLoS One.

[18] Erlwein O, Robinson MJ, Dustan S, Weber J, Kaye S (2011). DNA extraction columns contaminated with murine sequences.. PLoS One.

[19] Hue S, Gray ER, Gall A, Katzourakis A, Tan CP (2010). Disease-associated XMRV sequences are consistent with laboratory contamination.. Retrovirology.

[20] Sfanos KS, Aloia AL, Hicks JL, Esopi DM, Steranka JP (2011). Identification of replication competent murine gammaretroviruses in commonly used prostate cancer cell lines.. PLoS One.

[21] Paprotka T, Delviks-Frankenberry KA, Cingoz O, Martinez A, Kung HJ (2011). Recombinant origin of the retrovirus XMRV.. Science.

[22] Oakes B, Tai AK, Cingoz O, Henefield MH, Levine S (2010). Contamination of human DNA samples with mouse DNA can lead to false detection of XMRV-like sequences.. Retrovirology.

[23] Smith RA (2010). Contamination of clinical specimens with MLV-encoding nucleic acids: implications for XMRV and other candidate human retroviruses.. Retrovirology.

[24] Robinson MJ, Erlwein OW, Kaye S, Weber J, Cingoz O (2010). Mouse DNA contamination in human tissue tested for XMRV.. Retrovirology.

[25] Sakuma T, Hue S, Squillace KA, Tonne JM, Blackburn PR (2011). No evidence of XMRV in prostate cancer cohorts in the Midwestern United States.. Retrovirology.

[26] Raisch KP, Pizzato M, Sun HY, Takeuchi Y, Cashdollar LW (2003). Molecular cloning, complete sequence, and biological characterization of a xenotropic murine leukemia virus constitutively released from the human B-lymphoblastoid cell line DG-75.. Virology.

[27] Katzourakis A, Hue S, Kellam P, Towers GJ (2011). Phylogenetic analysis of MLV sequences from longitudinally sampled Chronic Fatigue Syndrome patients suggests PCR contamination rather than viral evolution.. J Virol.

[28] Switzer W, Jia H, Zheng H, Tang S, Garcia R (2011). Serologic and PCR testing of persons with chronic fatigue syndrome in the United States shows no association with xenotropic or polytropic murine leukemia virus-related virus.. Retrovirology.

[29] Katoh K, Toh H (2008). Recent developments in the MAFFT multiple sequence alignment program.. Brief Bioinform.

[30] Tamura K, Peterson D, Peterson N, Stecher G, Nei M (2011). MEGA5: Molecular Evolutionary Genetics Analysis using Maximum Likelihood, Evolutionary Distance, and Maximum Parsimony Methods.. Mol Biol Evol.

